# Full Familiarisation Is Not Required for the Self-Paced 1 km Treadmill Walk to Predict Peak Oxygen Uptake in Phase IV Cardiac Patients

**DOI:** 10.3390/clinpract14010025

**Published:** 2024-02-08

**Authors:** Mandy L. Gault, Mark E. T. Willems

**Affiliations:** Institute of Applied Sciences, University of Chichester, Chichester PO19 6PE, UK

**Keywords:** walking speed, heart rate, oxygen uptake, metabolic equivalent

## Abstract

Exercise is a recommended part of phase IV cardiovascular rehabilitation (CR). The 1 km treadmill walk test (1-KTWT) is a submaximal continuous exercise test to predict cardiorespiratory fitness in patients with cardiovascular disease. We examined physiological, metabolic and subjective responses in patients with cardiovascular disease with self-selected, unchanging walking speed for two 1-KTWTs. Fifteen men (age: 65 ± 9 yr, height: 174 ± 5 cm, body mass: 86 ± 17 kg, BMI: 28.5 ± 5.5 kg·m^−2^, body fat%: 27.7 ± 7.5%, 10 on beta-blockers) were recruited from phase IV CR groups in the United Kingdom. Participants established a self-selected walking speed for the 1-KTWT and performed the 1-KTWT on separate days with recording of physiological responses to predict $\dot{V}$O_2peak_ with equations. For the two 1-KTWTs, no differences existed for walking speed, mean and maximal heart rates, oxygen uptake, predicted $\dot{V}$O_2peak_ (1st 1-KTWT (range: 41–78% $\dot{V}$O_2peak_, 95%CI, 53–65; 2nd 1-KTWT range: 43–78% $\dot{V}$O_2peak_, 95%CI, 52–65) and rating of perceived exertion. In phase IV cardiac patients, the 1-KTWT with self-selected, unchanging walking speed can be used for $\dot{V}$O_2peak_ prediction without the need for a full familiarisation. The self-selected constant walking speed for the first 1-KTWT can be used to support nonsupervised physical activity for phase IV CR patients.

## 1. Introduction

Cardiovascular rehabilitation (CR) is defined by the British Association of Cardiovascular Prevention and Rehabilitation (BACPR) as “The co-ordinated sum of activities required to influence favourably the underlying cause of cardiovascular disease, as well as to provide the best possible physical, mental and social conditions, so that the patients may, by their own efforts, preserve or resume optimal functioning in their community and through improved health behaviour, slow or reverse progression of disease” [1]. Exercise is a key CR activity with the recommendation that it should be delivered with a patient-centred approach to maximise early uptake and long-term adherence [1]. In the United Kingdom, CR for phase III (outpatient) and phase IV (community) patients offers exercise from no earlier than 6 weeks after the event/diagnosis, with earlier phases promoting physical activity, exercise and a reduction in sedentary time [1,2]. More specifically, phase III aims to educate, monitor and supervise cardiac patients on a wide range of exercise modalities. Subsequently, the transfer of cardiac patients to phase IV (community) is based on (1) clinical stability, (2) ability to monitor and regulate a moderate–vigorous activity intensity, and (3) ability to recognise their optimum level of exercise intensity to reduce the risk of exertion-related events.

Cochrane Reviews highlighted the importance of aerobic training for individuals living with coronary heart disease (CHD) [3,4]. In a Canadian General Hospital, CHD patients with stable medical therapy were registered for a programme of moderate-intensity continuous exercise (n = 7, 3 d/wk for 12 weeks at 60–80% of peak heart rate) or stair-based high-intensity interval exercise (n = 9, 3 d/wk 3 × 6 flights for 12 weeks), which improved the capillary-to-fibre perimeter index by more than 10% in type I and II muscle fibres and indicative of improved microvascular circulation [5]. In a similar cohort of patients with CHD, Dunford et al. [6] examined moderate-intensity continuous exercise (n = 9, 30 min at 60–80% of peak heart rate) or stair-based high-intensity interval exercise (n = 9, 3 bouts 6 flights of stairs at self-selected vigorous intensity) for 4 weeks with six supervised structured exercise sessions and eight weeks of ~24 unsupervised exercise sessions. Both exercise programs were able to increase peak oxygen uptake (i.e., cardiorespiratory fitness, CRF) by more than 15% established with symptom-limited incremental cardiopulmonary exercise stress tests [6]. In general, CRF reflects the coordinated action of the respiratory and cardiovascular systems to meet the oxygen demand of muscle activity during physical exercise [7]. In nonclinical cohorts, CRF can provide an indication of the biological adaptations to a variety of physical training programs [8]. However, in clinical cohorts such as patients with CHD, CRF is an indication of disease-induced biological adaptations, pathophysiology, and a lower health status. Therefore, CRF in cardiac patients is not only important to assess disease severity but will also allow for examination of the progress of patients during CR [9].

CRF can be determined using maximal and submaximal exercise tests. The 1 km treadmill walk test (1-KTWT) is a submaximal exercise test to predict CRF in patients living with cardiovascular disease [10]. Such tests are more appropriate outside the clinical environment (community, phase IV) than maximal tests for which supervision by health care professionals is recommended. In addition, not only is it difficult to achieve a maximal effort from a cardiac patient [11], but submaximal tests are also safer and more applicable to activities of daily living that rarely require maximum effort [10]. Familiarisation of the testing procedures for a submaximal treadmill walking test is usually implemented to enable control over confounding influences and ensure that the observed changes are related to rehabilitation and not merely due to enhanced confidence with walking on a treadmill [12]. However, familiarisation takes time and resources. It is not known whether a self-selected walking speed from the first 1-KTWT may be used to support nonsupervised physical activity for patients with cardiovascular disease, excluding the need for a first formalised full familiarisation session.

The ability of phase IV cardiac patients to self-select the preferred exercise intensity during a submaximal walking test, such as the 1-KTWT, that would align to exercise recommendations is not known. The recommendations for exercise and physical activity for obtaining health benefits for individuals with and without cardiovascular disease provide guidance on the required intensity, exercise duration and exercise frequency [13,14]. Tracking of some specific physiological (e.g., heart rate) and subjective responses (i.e., rating of perceived exertion) may guide individualised exercise or physical activity, providing the patient-centred approach indicated by the BACPR to maximise long-term adherence [1]. Studies in healthy cohorts have assessed whether the self-selected intensity or preferred intensity was sufficient in meeting the exercise recommendations [15]. For example, the self-selected walking intensity for a typical nontreadmill walk by healthy habitual walkers (n = 29 (22 females), age: 36 ± 9 yr) met the required intensity of moderate-intensity exercise (i.e., 5.2 ± 1.2 metabolic equivalent) [15]. Other studies examined the effects of fitness and body weight on the preferred treadmill walk intensity [16], activity level on 20 min self-selected cycling power output [17] and exercise mode and gender on energy expenditure at self-selected intensities [18]. Decision making on self-selected exercise intensity has also been examined in clinical cohorts, e.g., Parkinson’s disease [19], adolescents with obesity [20] and older women with hypertension [21].

In Chiaranda et al. [10], walking speed was adjusted every 2 min during the 1-KTWT in male cardiac patients to achieve the speed for perceiving an exercise intensity of 11 to 13 on the 6–20 Borg scale. The ability of phase IV male cardiac patients to self-select a constant walking speed for the 1-KTWT and whether that speed would provide identical physiological and metabolic responses in repeated testing is not known. Therefore, the aim of the present study was to examine in phase IV male cardiac patients the repeatability of the self-selected intensity and unchanging walking speed on the physiological and metabolic responses of the 1-KTWT.

## 2. Materials and Methods

### 2.1. Participants and Ethical Approval

Fifteen men (age: 65 ± 9 yr, height: 174 ± 5 cm, body mass: 86 ± 17 kg, BMI: 28.5 ± 5.5 kg·m^−2^, and body fat%: 27.7 ± 7.5%) were recruited from phase IV CR groups in the Chichester area of the United Kingdom in response to flyers and posters distributed to phase IV exercise instructors. Before obtaining written informed consent from the participants to take part in the study, (1) a preparticipation health screening questionnaire was completed covering the inclusion and exclusion criteria (see below), (2) verbal understanding was obtained from the participants for the expectations and requirements of the study in the participant information sheet, and (3) approval for patient participation was obtained from a general practitioner. The study was approved by the University of Chichester Research Ethics Committee (code: 1516_33, approval date: 18 February 2016).

For study inclusion, participants were allowed to be classified with low risk (i.e., uncomplicated myocardial infarction, no left ventricular dysfunction, no exercise-induced ischaemia, no complex arrhythmias at rest or exercise and good exercise tolerance) and intermediate risk (i.e., mild-to-moderately reduced left ventricular function, ejection fraction: 31–49%, moderate exercise tolerance and exercise-induced myocardial ischemia) [22], or Classes I (i.e., no symptoms and limitations in normal physical activity) and II (mild symptoms and slight limitation during ordinary activity) of the New York Heart Association functional classification. Ten participants were taking beta-blockers but were allowed to participate following general practitioner approval [23]. Participants with reported low self-efficacy towards exercise were excluded [24].

The cardiac conditions and interventions of the participants are provided in Table 1. The medications at the time of the present study were recorded [25].

### 2.2. Experimental Visits

Participants attended the laboratory on 2 occasions at the same time of day, with the visits separated by at least one week. Before each visit, the participants were instructed not to consume alcohol and not to perform any strenuous exercise for 24 h. On the day of testing, participants were instructed not to consume caffeine-containing drinks and be at least 3 h postprandial on arrival [26]. On arrival, verbal confirmation of the participants was obtained for adherence to the instruction. The procedures for sessions one and two were similar with measurements of body mass (Seca Model 880, Seca, Birmingham, UK), height (Holtain Ltd., Crymycg, UK), and body composition (Tanita BC418, Maeno-cho, Tokyo, Japan) with removal of footwear and outer clothing and following instrument use instructions and measurement of the one-metabolic equivalent (1-MET). The rate pressure product (RPP, systolic blood pressure × heart rate) was only determined in session one. In the first visit, participants rested while in a beach chair position on a portable treatment table for 15 min during which expired air was collected and analysed with a breath-by-breath metabolic system (Jaegar Oxycon Pro, Carefusion, Basingstoke, UK) to assess the 1-MET. Cardiovascular parameters, i.e., blood pressure on the right arm and heart rate were recorded (Omron 705IT, Medisave, UK) at the end of the 15 min rest period to allow for the calculation of RPP. Subsequently, participants were allowed familiarisation with treadmill (Woodway Ergo ELG 70, Cranlea & Co., Birmingham, UK) walking for up to 15 min [12]. The starting speed was 1.5 km∙h^−1^, with the participants able to increase the walking speed gradually to the speed that they felt they could maintain for 20 min. Participants were continuously in communication with the researcher to allow for the emphasis that the aimed walking speed should be one that they could maintain for 20 min. This speed was considered the self-selected walking speed [27]. Subsequently, the participants completed the 1 km treadmill walk test (1-KTWT) [10]. The 1-KTWT allows for the prediction of peak oxygen uptake (see below for equations). During the 1-KTWT, the participants were allowed to lightly grip the handrails for balance but not support [10]. During the 1-KTWT, expired air was continuously sampled and analysed using the breath-by-breath metabolic system with continuous heart rate recording (RS400 Polar Electro, Finland) and the rating of perceived exertion (6–20 Borg scale) taken every 2 min and at time of completion of the 1-KTWT. The Jaegar Oxycon Pro was calibrated for volume and fractions of oxygen and carbon dioxide on each occasion in line with the manufacturer’s guidelines. In the second visit, all the procedures for treadmill walking and measurements were repeated.

The following equations from Chiaranda et al. [10] are from a study with cardiovascular patients that were not receiving βeta-blockers (Equation (1)) and receiving βeta-blockers (Equation (2)) and were used for the prediction of $\dot{V}$O_2peak_ (mL∙kg^−1^∙min^−1^).

(1) $$\dot{V}O_{2}=46.11+4.41\times mean\;walking\;speed-0.40\times BMI\;-0.30\times age-0.11\times {HR}_{max}$$

(2) $$\dot{V}O_{2peak}=33.42+2.79\times mean\;walking\;speed-0.49\times BMI-0.14\times age$$

where the mean walking speed is the self-selected walking speed during the 1-KTWT, BMI is the body mass index and HR_max_ is the age-predicted maximum heart rate.

### 2.3. Statistical Analysis

Statistical analyses were completed using Graphpad Prism (version 5 for Windows, Graphpad Software, San Diego, CA, USA). Normal distribution of the data was verified with the Kolmogorov–Smirnov test. Physiological, metabolic and subjective responses for the 1st and 2nd 1-KTWTs were analysed using paired samples student *t*-tests and pearson correlation coefficients were calculated. Repeatability of the predicted $\dot{V}$O_2peak_ for the for the 1st and 2nd 1-KTWTs was also analysed using Bland–Altman analysis with 95% limits of agreement. The data are reported as the mean ± SD, range and 95% confidence intervals. Significance was accepted at *p* ≤ 0.05.

## 3. Results

### 3.1. Baseline Observations at Rest

In the first session with participants resting in a beach chair position, the following physiological and cardiovascular parameters were recorded, i.e., 1-MET: 3.18 ± 0.65 mL·kg^−1^·min^−1^ (range: 1.92–3.99, 95%CI, 2.82–3.54), heart rate: 59 ± 11 bpm (range: 38–76, 95%CI, 53–65), systolic blood pressure: 132 ± 12 mmHg (range: 115–148, 95%CI, 125–138), diastolic blood pressure: 77 ± 7 mmHg (range: 64–87, 95%CI, 74–81) and rate pressure product: 7703 ± 1398 mmHg·bpm (range: 5358–9500, 95%CI, 6929–8477).

### 3.2. Walking Observations: Self-Selected Walking Speed and Walking Time

**Figure 1:**
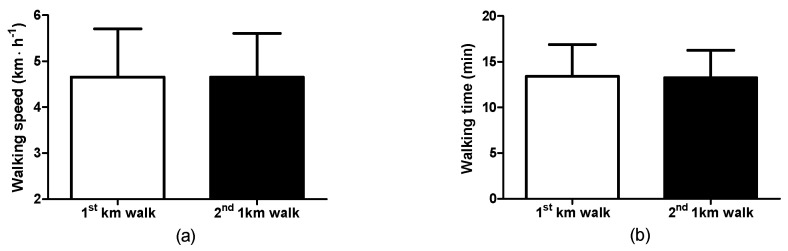
1-KTWT speeds (**a**) and walking time (**b**) for the 1st and 2nd sessions. Data are the mean ± SD.

The participants performed two 1-KTWTs in separate sessions. The self-selected walking speeds for the 1st and 2nd 1-KTWTs were not different (Figure 1a), i.e., 1st 1-KTWT (range: 2.5–6.6 km·h^−1^, 95%CI, 4.1–5.2) and for the 2nd 1-KTWT (range: 2.9–6.2 km·h^−1^, 95%CI, 4.1–5.2). Four of the fifteen participants had exactly the same walking speed for the two sessions. The correlation coefficient between the speeds for the 1st and 2nd 1-KTWTs was 0.980 (*p* < 0.01). Accordingly, there were no differences for the walk time (Figure 1b), i.e., 1st 1-KTWT (range: 9.05–23.34 min, 95%CI, 11.47–15.33) and for the 2nd 1-KTWT (range: 9.42–20.35 min, 95%CI, 11.59–14.92).

### 3.3. Walking Observations: Physiological and Cardiovascular Responses

**Figure 2:**
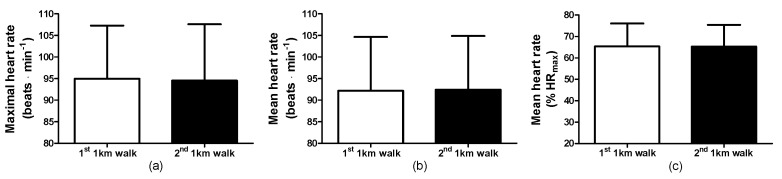
Maximal heart rates (**a**), mean heart rates (**b**), and the mean heart rate as a percentage of the age-predicted maximal heart rate (**c**) for the 1-KTWT in the 1st and 2nd session. Data are the mean ± SD.

The maximal heart rates during the 1st and 2nd 1-KTWTs were not different (Figure 2a, i.e., 1st 1-KTWT (range: 80–122 beats·min^−1^, 95%CI, 88–102) and for the 2nd 1-KTWT (range: 77–126 beats·min^−1^, 95%CI, 87–102). In addition, the mean heart rates during the 1st and 2nd 1-KTWTs were not different (Figure 2b), i.e., 1st 1-KTWT (range: 76–119 beats·min^−1^, 95%CI, 85–99) and for the 2nd 1-KTWT (range: 75–115 beats·min^−1^, 95%CI, 85–99). As a consequence, the mean heart rate as a % of the age-predicted maximum heart rate were not different (i.e., 1st 1-KTWT: 65 ± 11% (range: 48–90%, 95%CI, 59–71) and for the 2nd 1-KTWT: 65 ± 10% (range: 49–87%, 95%CI, 60–71%) (Figure 2c) and the predicted $\dot{V}$O_2peak_ during the 1st and 2nd 1-KTWTs were not different, i.e., 1st 1-KTWT (range: 17.9–31.9 mL∙kg^−1^∙min^−1^, 95%CI, 21.6–26.6) and for the 2nd 1-KTWT (range: 17.9–31.9 mL∙kg^−1^∙min^−1^, 95%CI, 21.7–26.4).

**Figure 3:**
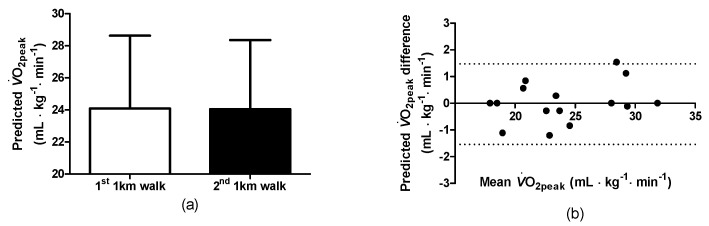
Predicted $\dot{V}$O_2peak_ during the 1st and 2nd 1-KTWTs (**a**); Bland–Altman for the predicted $\dot{V}$O_2peak_ (**b**). $\dot{V}$O_2_, oxygen uptake. Data in (**a**) are the mean ± SD.

**Figure 4:**
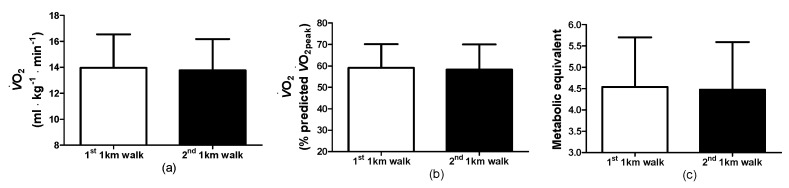
Oxygen uptake (**a**), %$\dot{V}$O_2peak_ (**b**) and the metabolic equivalents (**c**) for the 1st and 2nd 1-KTWTs. $\dot{V}$O_2_, oxygen uptake. Data are the mean ± SD.

For the predicted $\dot{V}$O_2peak_ during the 1st and 2nd 1-KTWTs (Figure 3a), the correlation was high (0.986) (*p* < 0.01) and the Bland–Altman plot indicated agreement for the two 1-KTWT’s (Figure 3b). During the walks, the oxygen uptakes during the 1st and 2nd 1-KTWTs were not different (Figure 4a), i.e., 1st 1-KTWT (range: 10.0–21.6 mL∙kg^−1^∙min^−1^, 95%CI, 12.5–15.4) and for the 2nd 1-KTWT (range: 9.1–19.0 mL∙kg^−1^∙min^−1^, 95%CI, 12.4–15.1). As a consequence, the %$\dot{V}$O_2peak_ during the 1st and 2nd 1-KTWTs were not different (Figure 4b), i.e., 1st 1-KTWT (range: 41–78%$\dot{V}$O_2peak_, 95%CI, 53–65) and for the 2nd 1-KTWT (range: 43–78%$\dot{V}$O_2peak_, 95%CI, 52–65). In addition, the metabolic equivalents during the 1st and 2nd 1-KTWTs were not different (Figure 4c), i.e., 1st 1-KTWT (range: 2.9–7.3 MET, 95%CI, 3.9–5.2) and for the 2nd 1-KTWT (range: 2.8–7.1 MET, 95%CI, 3.9–5.1).

### 3.4. Walking Observations: Rating of Perceived Exertion

**Figure 5:**
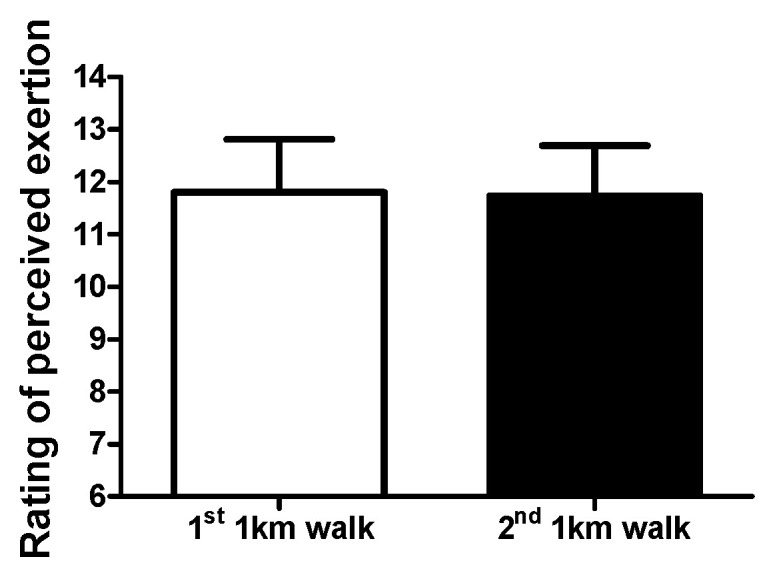
Rating of perceived exertion during the 1st and 2nd 1-KTWTs. Data are the mean ± SD.

The rating of perceived exertion during the 1st and 2nd 1-KTWTs were not different (Figure 5), i.e., 1st 1-KTWT (range: 9–13, 95%CI, 11–12) and for the 2nd 1-KTWT (range: 10–13, 95%CI, 11–12).

## 4. Discussion

For a cohort of adults living with cardiovascular disease, novel information is provided on whether a first session for the 1-KTWT can be used to predict the cardiovascular fitness parameter $\dot{V}$O_2peak_. If that would not be the case, then a second session for the 1-KTWT would result in different predicted $\dot{V}$O_2peak_ values. However, the agreement in predicted $\dot{V}$O_2peak_, physiological and subjective responses provide evidence that the self-selected intensity in the first session for the 1-KTWT in a cohort of phase IV cardiovascular patients can be used for the prediction of $\dot{V}$O_2peak_.

Studies on the effects of an unfamiliar exercise task will commonly incorporate familiarisation session(s) to allow for acclimation to exclude potential learning effects that may affect the study outcomes. Treadmill walking for the first time is an unfamiliar exercise task and provides different sensorimotor responses than over ground walking [28]. The need for familiarisation was recognised in 2006 by Van de Putte et al. [29] with the recommendation to allow 10 min of treadmill walking before measurement of knee kinematic and spatio-temporal data. In addition, older males and females (age: 51–80 years) without neurological or locomotor pathology required at least 6 min of treadmill walking to ensure stability in 26 walking parameters [30]. In the present study, phase IV cardiovascular patients were allowed ~15 min of acclimation with treadmill walking to self-select a walking speed to enable them to complete a 1-KTWT in two separate sessions.

The treadmill walking speeds for the 1st and 2nd 1-KTWTs in our cohort were 4.6 ± 1.0 and 4.7 ± 1.0 km·h^−1^. In healthy older individuals (66–80 years), Malatesta et al. [31] observed that the preferred treadmill walking speed (i.e., 4.7 ± 0.5 km·h^−1^) was slower than the preferred overground indoor walking speed (5.2 ± 0.6 km·h^−1^). However, it needs to be noted that the preferred treadmill walking speed in Malatesta et al. [31] was not selected for the performance of a 1-KTWT. A self-paced supervised community-based walking programme in New Zealand had cardiac patients complete one-mile walks in an outdoor environment, eliciting similar walking velocities (4.6 ± 0.6 km·h^−1^) and heart rates (106 ± 14 beats·min^−1^) in week 1 to the present study and Faulkner et al. [32]. In 1442 male outpatients with CVD, Grazzi et al. [33] observed a walking speed and heart rate for a perceptually regulated 1-KTWT of 4.4 ± 1.1 km·h^−1^ and 95 ± 14 beat·min^−1^ with predicted $\dot{V}$O_2peak_ of 23.9 ± 4.6 mL·kg^−1^·min^−1^. Additionally, Chiaranda et al. [34], in 1491 male cardiovascular patients, the walking speed during the 1-KTWT was 4.2 ± 1.0 km·h^−1^ and 96 ± 14 beats·min^−1^ with predicted $\dot{V}$O_2peak_ of 22.7 ± 5.6 mL·kg^−1^·min^−1^. Overall, the self-selected walking speeds and heart rates in our phase IV cardiovascular patients are comparable to what has been reported in the literature. However, the methodology of the self-selected walking speed in our cohort was different than reported in other studies. In those studies, the walking test started with subjects walking on a level gradient at a walking speed of 2.0 km·h^−1^ with subsequent increases of 0.3 km·h^−1^ each 30 s up to a moderate walking speed, corresponding to a perceived exertion intensity of 11 to 13 of 20 using the RPE scale [10]. The study by Chiaranda et al. [10] which provided the equations to predict $\dot{V}$O_2peak_ did not have a constant speed. In the present study, no structured guidance as in Chiaranda et al. [10] was provided. Nevertheless, the predicted $\dot{V}$O_2peak_ of ~24 mL∙kg^−1^∙min^−1^ in our phase IV cardiovascular patients is similar to the predicted $\dot{V}$O_2peak_ of 23.9 ± 4.6 mL·kg^−1^·min^−1^ in Grazzi et al. [33] and 22.7 ± 5.6 mL·kg^−1^·min^−1^ in Chiaranda et al. [34], both in male cohorts.

In a cohort of male outpatients (n = 64) with stable cardiovascular disease, the 1 km treadmill walking was used to predict changes in peak oxygen uptake from an 8 wk walking programme [35]. The walking programme consisted of supervised and unsupervised walking sessions. In Raisi et al. [35], the measured peak oxygen uptake from a maximal treadmill test was similar to the predicted equation-calculated peak oxygen uptake. Therefore, the 1 km treadmill walking test was considered useful to predict changes in peak oxygen uptake from interventions that are part of cardiovascular rehabilitation programs [35]. From the same research group, a recent study by Raisi et al. [36] used the predicted peak oxygen uptake of the 1 km treadmill walking test for risk stratification in female cardiovascular patients. It needs to be noted that an equation to predict peak oxygen uptake is now also available for female patients with cardiovascular disease [37]. The self-selected walking speed combined with oxygen uptake measurement at rest and during the walk allows for the consideration of the exercise intensity expressed as a metabolic equivalent. The 1-MET shows substantial variation in nonclinical and clinical cohorts [26,38]. In the present study, the 1-MET value was slightly higher and recorded with more variation than reported in the supine position in a large cohort of men with coronary heart disease and BMI > 25.0 kg·m^−2^ (MET was a $\dot{V}$O_2_ value of 2.58 ± 0.4 mL∙kg^−1^∙min^−1^) [38]. Nevertheless, on the basis of the individual 1-MET values, our cohort was walking the 1-KTWT with a metabolic equivalent for the 1st and 2nd walks of 4.6 ± 1.2 (range: 2.9–7.3) and 4.5 ± 1.1 (range: 2.8–7.1). From the 15 participants, 13 selected a walking speed that provided a MET value between 3 and 6 MET (i.e., categorised as moderate intensity) for the 2nd 1-KTWT. Participants were able to be consistent in their choice of self-selected walking speed, and the choice also allowed for the completion of the 1-KTWT with a moderate intensity. This observation has application in the health promotion of phase IV cardiac patients, as it demonstrates their ability to choose an exercise intensity that is known to provide health benefits when regular exercise sessions with that intensity would be part of a walking programme based on the preferred treadmill walking speed and not adopting excessive exercise [39].

## 5. Conclusions

Familiarisation for uncommon exercise tasks is provided for participants to become accustomed with completion of the task and associated testing procedures. However, for studies with large clinical cohorts, familiarisation is labour- and time-intensive. The observations of the present study allow for the conclusion that phase IV male cardiac patients do not need a full familiarisation session for a 1-KTWT. Future studies with phase IV cardiac patients may want to adopt our protocol when the aim is to examine the effects of interventions on the physiological and metabolic responses of the 1-KTWT. In addition, a future study may want to address the familiarisation of the 1-KTWT in a female cohort of cardiovascular patients.

## Figures and Tables

**Table 1:** Cardiac conditions and interventions of participants.

Cardiac Condition/Intervention	n
Myocardial infarction and percutaneous intervention	6
Angina and percutaneous intervention	1
Valve replacement surgery	3
Myocardial infarction and coronary artery bypass graph surgery	3
Pacemaker	1
Heart failure (class I/II)	1

## Data Availability

Data are available upon reasonable request.

## References

[1] Jones J., Furze G., Buckley J., Jones J., Furze G., Buckley J., Sheppard G. (2020). Cardiovascular Disease Prevention and Rehabilitation. Cardiovascular Prevention and Rehabilitation in Practice.

[2] National Audit of Cardiac Rehabilitation Annual Statistical Report 2017. https://www.bhf.org.uk/.

[3] Heran B.S., Chen J.M., Ebrahim S., Moxham T., Oldridge N., Rees K., Thompson D.R., Taylor R.S. (2011). Exercise-based cardiac rehabilitation for coronary heart disease. Cochrane Database Syst. Rev..

[4] Dibben G., Faulkner J., Oldridge N., Rees K., Thompson D.R., Zwisler A., Taylor R.S. (2021). Exercise-based cardiac rehabilitation for coronary heart disease. Cochrane Database Syst. Rev..

[5] Lim C., Dunford E.C., Valentino S.E., Oikawa S.Y., McGlory C., Baker S.K., Macdonald M.J., Phillips S.M. (2021). Both Traditional and Stair Climbing-based HIIT Cardiac Rehabilitation Induce Beneficial Muscle Adaptations. Med. Sci. Sports Exerc..

[6] Dunford E.C., Valentino S.E., Dubberley J., Oikawa S.Y., McGlory C., Lonn E., Jung M.E., Gibala M.J., Phillips S.M., MacDonald M.J. (2021). Brief Vigorous Stair Climbing Effectively Improves Cardiorespiratory Fitness in Patients with Coronary Artery Disease: A Randomized Trial. Front. Sports Act. Living.

[7] Ross R., Blair S.N., Arena R., Church T.S., Després J.P., Franklin B.A., Haskell W.L., Kaminsky L.A., Levine B.D., Lavie C.J. (2016). Importance of Assessing Cardiorespiratory Fitness in Clinical Practice: A Case for Fitness as a Clinical Vital Sign: A Scientific Statement from the American Heart Association. Circulation.

[8] Wenger H.A., Bell G.J. (1986). The interactions of intensity, frequency and duration of exercise training in altering cardiorespiratory fitness. Sports Med..

[9] Bruce R.A., Kusumi F., Hosmer D. (1973). Maximal oxygen intake and nomographic assessment of functional aerobic impairment in cardiovascular disease. Am. Heart J..

[10] Chiaranda G., Myers J., Mazzoni G., Terranova F., Bernardi E., Grossi G., Codecà L., Conconi F., Grazzi G. (2012). Peak oxygen uptake prediction from a moderate, perceptually regulated, 1-km treadmill walk in male cardiac patients. J. Cardiopulm. Rehabil. Prev..

[11] Weber K.T., Kinasewitz G.T., Janicki J.S., Fishman A.P. (1982). Oxygen utilization and ventilation during exercise in patients with chronic cardiac failure. Circulation.

[12] Wass E., Taylor N.F., Matsas A. (2005). Familiarisation to treadmill walking in unimpaired older people. Gait Posture.

[13] Franklin B.A., Eijsvogels T.M.H., Pandey A., Quindry J., Toth P.P. (2022). Physical activity, cardiorespiratory fitness, and cardiovascular health: A clinical practice statement of the American Society for Preventive Cardiology Part II: Physical activity, cardiorespiratory fitness, minimum and goal intensities for exercise training, prescriptive methods, and special patient populations. Am. J. Prev. Cardiol..

[14] Franklin B.A., Eijsvogels T.M.H., Pandey A., Quindry J., Toth P.P. (2022). Physical activity, cardiorespiratory fitness, and cardiovascular health: A clinical practice statement of the ASPC Part I: Bioenergetics, contemporary physical activity recommendations, benefits, risks, extreme exercise regimens, potential maladaptations. Am. J. Prev. Cardiol..

[15] Spelman C.C., Pate R.R., Macera C.A., Ward D.S. (1993). Self-selected exercise intensity of habitual walkers. Med. Sci. Sports Exerc..

[16] Pintar J.A., Robertson R.J., Kriska A.M., Nagle E., Goss F.L. (2006). The influence of fitness and body weight on preferred exercise intensity. Med. Sci. Sports Exerc..

[17] Dishman R.K., Farquhar R.P., Cureton K.J. (1994). Responses to preferred intensities of exertion in men differing in activity levels. Med. Sci. Sports Exerc..

[18] Kravitz L., Robergs R.A., Heyward V.H., Wagner D.R., Powers K. (1997). Exercise mode and gender comparisons of energy expenditure at self-selected intensities. Med. Sci. Sports Exerc..

[19] Kanegusuku H., Cucato G.G., Longano P., Okamoto E., Piemonte M.E.P., Correia M.A., Ritti-Dias R.M. (2022). Acute Cardiovascular Responses to Self-selected Intensity Exercise in Parkinson’s Disease. Int. J. Sports Med..

[20] Barros T.A.R., do Prado W.L., Tenório T.R.S., Ritti-Dias R.M., Germano-Soares A.H., Balagopal B.P., Hill J.O., Freitas-Dias R. (2021). Cardiovascular Effects of Aerobic Exercise with Self-Selected or Predetermined Intensity in Adolescents with Obesity. Pediatr. Exerc. Sci..

[21] Costa I.B.B., Schwade D., Macêdo G.A.D., Browne R.A.V., Farias-Junior L.F., Freire Y.A., Sócrates J., Boreskie K.F., Duhamel T.A., Caldas Costa E. (2019). Acute antihypertensive effect of self-selected exercise intensity in older women with hypertension: A crossover trial. Clin. Interv. Aging.

[22] Bethell H.J.N. (2006). Exercise-based cardiac rehabilitation. Medicine.

[23] Gordon N.F., Duncan J.J. (1991). Effect of beta-blockers on exercise physiology: Implications for exercise training. Med. Sci. Sports Exerc..

[24] Resnick B., Jenkins L.S. (2000). Testing the reliability and validity of the Self-Efficacy for Exercise scale. Nurs. Res..

[25] McInnis K.J., Balady G.J. (1994). Comparison of submaximal exercise responses using the Bruce vs modified Bruce protocols. Med. Sci. Sports Exerc..

[26] Byrne N.M., Hills A.P., Hunter G.R., Weinsier R.L., Schutz Y. (2005). Metabolic equivalent: One size does not fit all. J. Appl. Physiol..

[27] Gault M.L., Clements R.E., Willems M.E.T. (2012). Functional mobility of older adults after concentric and eccentric endurance exercise. Eur. J. Appl. Physiol..

[28] Rossignol S., Dubuc R., Gossard J.P. (2006). Dynamic sensorimotor interactions in locomotion. Physiol. Rev..

[29] Van de Putte M., Hagemeister N., St-Onge N., Parent G., de Guise J.A. (2006). Habituation to treadmill walking. Biomed. Mater. Eng..

[30] Meyer C., Killeen T., Easthope C.S., Curt A., Bolliger M., Linnebank M., Zörner B., Filli L. (2019). Familiarization with treadmill walking: How much is enough?. Sci. Rep..

[31] Malatesta D., Canepa M., Menendez Fernandez A. (2017). The effect of treadmill and overground walking on preferred walking speed and gait kinematics in healthy, physically active older adults. Eur. J. Appl. Physiol..

[32] Faulkner J., Gerhard J., Stoner L., Lambrick D. (2012). Self-Paced Walking within a Diverse Topographical Environment Elicits an Appropriate Training Stimulus for Cardiac Rehabilitation Patients. Rehabil. Res. Pract..

[33] Grazzi G., Mazzoni G., Myers J., Codecà L., Pasanisi G., Mandini S., Piepoli M., Volpato S., Conconi F., Chiaranda G. (2018). Determining the best percent-predicted equation for estimated VO_2_ peak by a 1-km moderate perceptually-regulated treadmill walk to predict mortality in outpatients with cardiovascular disease. J. Sci. Med. Sport..

[34] Chiaranda G., Myers J., Arena R., Kaminsky L., Sassone B., Pasanisi G., Mandini S., Mazzoni G., Grazzi G. (2021). Prognostic comparison of the FRIEND and Wasserman/Hansen peak VO_2_ equations applied to a submaximal walking test in outpatients with cardiovascular disease. Eur. J. Prev. Cardiol..

[35] Raisi A., Bernardi E., Myers J., Piva T., Zerbini V., Massotti S., Menegatti E., Caruso L., Mazzoni G., Grazzi G. (2023). Change in Peak Oxygen Uptake Predicted by the Moderate 1-km Treadmill Walking Test After Walking Training in Outpatients with Cardiovascular Disease. J. Cardiopulm. Rehabil. Prev..

[36] Raisi A., Piva T., Myers J., Lordi R., Zerbini V., Massotti S., Chiaranda G., Grazzi G., Mazzoni G., Mandini S. (2023). A Moderate Walking Test Predicts Survival in Women with Cardiovascular Diseases. Am. J. Prev. Med..

[37] Zerbini V., Raisi A., Myers J., Piva T., Lordi R., Chiaranda G., Mazzoni G., Grazzi G., Mandini S. (2021). Peak Oxygen Uptake Estimation From A Moderate 1-KM Treadmill Walk in Women with Cardiovascular Disease. J. Cardiopulm. Rehabil. Prev..

[38] Savage P.D., Toth M.J., Ades P.A. (2007). A re-examination of the metabolic equivalent concept in individuals with coronary heart disease. J. Cardiopulm. Rehabil. Prev..

[39] Williams P.T., Thompson P.D. (2014). Increased cardiovascular disease mortality associated with excessive exercise in heart attack survivors. Mayo Clin. Proc..

